# Bluetongue Virus Serotype 1 Outbreak in the Basque Country (Northern Spain) 2007–2008. Data Support a Primary Vector Windborne Transport

**DOI:** 10.1371/journal.pone.0034421

**Published:** 2012-03-30

**Authors:** Rodrigo García-Lastra, Iratxe Leginagoikoa, Jose M. Plazaola, Blanca Ocabo, Gorka Aduriz, Telmo Nunes, Ramón A. Juste

**Affiliations:** 1 Department of Animal Health, NEIKER-Tecnalia, Derio, Bizkaia, Spain; 2 Departamento de Desarrollo Rural, Diputación Foral de Gipuzkoa, Donostia, Gipuzkoa, Spain; 3 Departamento de Agricultura, Diputación Foral de Bizkaia, Bilbao, Bizkaia, Spain; 4 Faculdade de Medicina Veterinária, TU Lisbon, Lisbon, Portugal; Institute for Animal Health, United Kingdom

## Abstract

**Background:**

Bluetongue (BT) is a vector-borne disease of ruminants that has expanded its traditional global distribution in the last decade. Recently, BTV-1 emerged in Southern Spain and caused several outbreaks in livestock reaching the north of the country. The aim of this paper was to review the emergence of BTV-1 in the Basque Country (Northern Spain) during 2007 and 2008 analyzing the possibility that infected *Culicoides* were introduced into Basque Country by winds from the infected areas of Southern Spain.

**Methodology/Principal Findings:**

We use a complex HYSPLIT (Hybrid Single-Particle Lagrangian Integrated Trajectory) model to draw wind roses and backward wind trajectories. The analysis of winds showed September 28 to October 2 as the only period for the introduction of infected midges in the Basque Country. These wind trajectories crossed through the areas affected by serotype 1 on those dates in the South of the Iberian Peninsula. Additionally meteorological data, including wind speed and humidity, and altitude along the trajectories showed suitable conditions for *Culicoides* survival and dispersion.

**Conclusions/Significance:**

An active infection in medium-long distance regions, wind with suitable speed, altitude and trajectory, and appropriate weather can lead to outbreaks of BTV-1 by transport of *Culicoides imicola*, not only over the sea (as reported previously) but also over the land. This shows that an additional factor has to be taken into account for the control of the disease which is currently essentially based on the assumption that midges will only spread the virus in a series of short hops. Moreover, the epidemiological and serological data cannot rule out the involvement of other *Culicoides* species in the spread of the infection, especially at a local level.

## Introduction

Bluetongue (BT) is an arthropod-borne disease caused by a virus of the genus *Orbivirus*, the Bluetongue virus (BTV), which affects ruminant livestock such as cattle, sheep, and goats and wild ruminants such as deer, and camelids. Infected animals can show mild or no detectable clinical signs, but others can develop a clinical disease with signs ranging from fever, anorexia and weight loss, to nasal discharges, excessive salivation, edema of the lips, tongue and head, conjunctivitis, coronitis, lameness or abortions [1], [2]. There are at least 26 BTV serotypes vectored by different species of midges of the genus *Culicoides* spp. depending on the geographic area and climatic factors [3].

### Global distribution and recent emergence

Historically, BTV distribution across the world covered a broad band, approximately, between 40°N and 35°S, where BTV has been enzootic throughout sub-Saharan Africa and wide areas of Asia and the Middle East. In Europe, and before 1998, outbreaks in Cyprus, the Iberian Peninsula and Greece were caused by brief sporadic incursions from adjacent enzootic regions and only with a single BTV serotype involved [4]. However, between 1998 and 2005, five serotypes of BTV (serotypes 1, 2, 4, 9 and 16) have been continuously present in the Mediterranean Basin, including several member states of the EU [5], [6].

Thus, it seems that the global distribution of BTV infection has recently experienced an important change. Some authors have proposed that climate change is partially responsible for this modification in the BTV global distribution [7], [8]. This fact could be explained by its impact on the vectorial capacity of resident *Culicoides* populations in previously virus-free regions such as much of the Mediterranean Basin. Nevertheless, the epidemiology of recent emergence of BTV-8 in Northern Europe would appear to be different from the spread of several BTV serotypes throughout the Mediterranean countries [4]. Although, in the first stages of the epidemic, transmission seemed to be attributable mainly to *Culicoides imicola*, the participation of novel vectors (*C. obsoletus*, *C. scoticus, C. pulicaris and C. newsteadi*) in regional spread of the virus was quickly confirmed [9], [10].

### Bluetongue in Spain

In Spain, the epidemiologic situation of BT changed significantly in the recent years. OIE have considered Spain free of serotype 2 since December 2002. In October 2004, the surveillance program revealed the circulation of BTV serotype 4 in the Southern area of the country. New outbreaks were detected in 2005 and 2006. The vaccination strategy and the preventive measures implemented to control the disease led to the absence of BTV-4 circulation during 2007 and 2008. In March 2009, the whole country was officially declared “free” of BTV-4. This situation has remained until the end of 2010.

In October 2008 all national territory was certificated as a BTV-1 and BTV-8 Restricted Zone (excluding the Balearics and Canarias Islands).

Recent outbreaks of serotypes 1 and 4 in the last year have changed the status again. The Southern provinces of Cadiz, Huelva, Malaga and the south of the province of Sevilla was declared as a BTV-1,4,8 Restricted Zone [11].

### Objective

The Basque Country was considered as a zone of low risk for BT in 2007. However, in November 2007 a BTV-1 outbreak appeared in Oiartzun (Gipuzkoa province) and quickly spread to neighbouring zones reaching Navarre and the Pyrenees Atlantiques in France in the following weeks. This prompted two studies about the vectors, one of which reported the presence of *Culicoides imicola* in the area [12], [13]. Since this finding could suggest that rather than a ruminant carrier or transportation of infected midges in vehicles or containers, the infected vector could have reached the Basque Country by the way of airborne swarms, we have examined the archived information on outbreaks and livestock movements together with the meteorological records and especially the winds in order to see the likelihood of this hypothesis.

## Materials and Methods

### Serological data

The evolution of the infection at the first stages was evaluated by serological investigation of herds with animals showing possible clinical signs of BT in the province of Gipuzkoa. The analyses were carried out in the Microbiology and Immunology Laboratory of NEIKER (Basque Institute for Agricultural Research and Development). The serologic test was a commercial blocking ELISA (INGEZIM BTV COMPAC, INGENASA, Spain) for the detection of antibodies against BTV.

In order to establish the likelihood of a single broad landing area of infected swarms versus a single point terrestrial arrival, we compared the proportion of herds with over 50% bluetongue seropositive animals in the first municipalities with clinical cases. This proportion in three municipalities which had herds with 100% of positive animals and were territorially contiguous was compared with the proportion in the rest of municipalities in the province of Gipuzkoa. The statistical analysis used for this comparison was the Fisher exact probability test included in the FREQ procedure of the SAS statistical package (SAS Institute 9.1.Cary, NC, USA).

### Entomological data

Data on the capture of *Culicoides* midges were obtained from the trapping carried out by the Livestock Services of each one of the three Basque territories, within the framework of the National Bluetongue Entomological Surveillance Program, as well as published information [13].

### Analysis of winds and wind trajectories

In order to assess the likelihood of a possible long-range airborne transport over Spain, we assumed that the *Culicoides* midges, presumably of the longtime recognized main African-European vector *C. imicola* associated with the bluetongue outbreaks in the southern Iberian Peninsula, behave essentially as the dust particles for which the atmospheric dispersion models were originally developed [14]–[16]. To this end, we analyzed the frequency and directions of winds using archived data to draw wind roses and backward trajectories with the HYSPLIT model (Hybrid Single-Particle Lagrangian Integrated Trajectory) available online [17]. We choose this model because it has already been used for Culicoides and other arthropod dispersal studies, [18]–[21], is readily available in the Internet and easy to use. It is provided by the U.S. National Oceanic and Atmospheric Administration's Air Resources Laboratory (NOAA-ARL), and uses a calculation method that is a hybrid between Eulerian and Lagrangian approaches to compute simple air-parcel trajectories to complex simulations, by using archived data. The application uses discrete measurements to produce a continuous dataset by the HYSPLIT model. The model can run on any datum-point on the globe, and simulate trajectories forward or backward to that point, at different heights and dates [22]. The model was calculated on GDAS (Global Data Assimilation System) data. This system is based on meteorological data which are measured four times a day. The data are provided in a resolution grid of 80 km×80 km.

**Figure 1:**
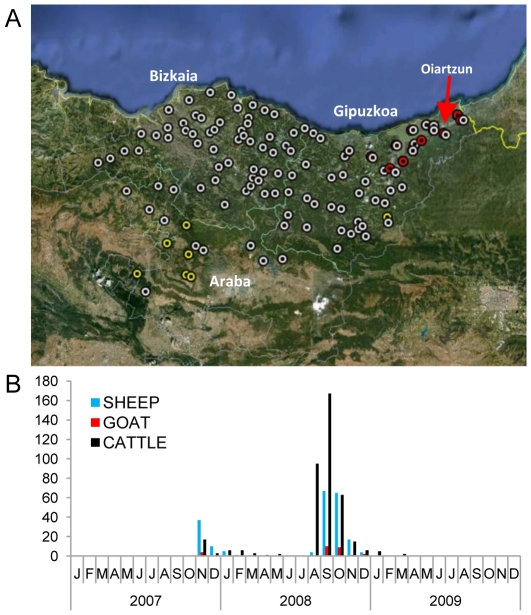
BTV-1 Basque Country outbreaks (2007–2009). A. Situation of the three provinces of the Basque Country (Bizkaia, Gipuzkoa and Araba). Circle marks show the 2007 (red), 2008 (white) and 2009 (yellow) outbreak locations. The red arrow shows the first outbreak in the Basque Country (Oiartzun, Gipuzkoa). B. Outbreak records by month for 2007–2009 periods.

We considered a minimum period of 4 weeks for travel and arrival of infected midges, feeding on susceptible hosts, development of viremia in these hosts (source of infection for new midges and radial short-distance spread), development of symptoms in susceptible hosts and detection-reporting of symptoms by the farmer or veterinarian [23]. Thus, we recovered daily frequencies and directions of winds since July 1 to October 21, 2007 from the first Northern outbreak in Oiartzun (Gipuzkoa, Basque Country) on 2 November, 2007 (Figure 1). Backward wind trajectories were obtained for the dates with predominant southerly winds at 10, 500, and 1000 meters above the ground level from Oiartzun, with a length of 72 hours and a time-step of 6 hours in each section. The trajectories were allowed to travel in the three dimensions throughout the atmosphere.

### Meteorological data

The HYSPLIT model dumped weather data along the backward trajectories. Pressure, terrain height, ambient temperature, rainfall and relative humidity data were collected in order to be analyzed together with wind and epidemiological information.

The weather data in the Basque Country in these dates were obtained from the Jaizkibel weather station near Oiartzun [24].

### Epidemiological data

Outbreaks information was recorded from the Spanish Ministry of the Environment and Rural and Marine Affairs and the regional authorities of the Basque Country. Mortality and morbidity rates were calculated for each species and years of the epidemics. Clinical signs and epidemiological data were recorded by a survey to affected farmers.

## Results

### BTV-1 outbreak in Basque Country

North African BTV-1 outbreaks in 2006 arrived to Andalusia, in the Southeast of the Iberian Peninsula in July 2007. The infection extended during the summer to neighboring provinces of Extremadura and Castile-La Mancha. Finally, BTV-1 arrived in Northern Spain (Basque Country, Navarre and Pyrenees Atlantiques in France). In November 2007, BTV-1 was first detected in Oiartzunaldea valley (province of Gipuzkoa), thus constituting the first outbreak in the Basque Country (Figure 1). Six specimens of *Culicoides imicola* were detected in both of the localities most affected by the outbreak of BTV-1 [13].

The 61 BT outbreaks in the Basque Country in 2007 were detected in Gipuzkoa, mainly in sheep flocks (n = 39) but also in cattle herds (n = 12), one goat flock and 9 mixed farms (sheep, goat and/or cattle in the same farm). In November 2007 a massive sheep vaccination program began in the three Basque Country provinces as well as in the rest of Spain. Vaccines were provided by the Ministry of the Environment and Rural and Marine Affairs. This vaccination program was limited to sheep due to limited vaccine production. Only 19 outbreaks were detected in winter and 3 in spring 2008, all of them in Gipuzkoa.

Cattle vaccination was started in spring of 2008, but it had not reached 100% of the census at the time of emergence of the new BTV1 outbreak in August 2008. Interestingly, two specimens of *Culicoides imicola* were also collected in July 23 and August 6, 2008 in the samplings realized in Gipuzkoa in the National Bluetongue Entomological Surveillance Program. This new peak of the epidemic began in cattle of Bizkaia and arrived to Araba and Gipuzkoa in September 2008. The BTV serotype 1 affected cattle herds (n = 307) mainly in Bizkaia (200 of the 307) probably due to the lack of vaccination of a great part of the cattle in this province. It was also detected in sheep flocks (n = 103), goat flocks (n = 5) and mixed herds (n = 63) of the three territories.

Finally, between January and March 2009, 7 new outbreaks (6 in Araba and 1 in Gipuzkoa) were detected, all of them in cattle. After that, no more BTV-1 outbreaks were detected in the Basque Country.

### Effects of BTV-1 outbreak

BTV-1 epidemics in Basque Country caused 546 outbreaks during the 2007 to 2009 period: 324 in cattle, 142 in sheep, 6 in goats and 74 in mixed farms [25]. Morbidity and mortality data collected from regional animal health authorities are shown in Table 1.

**Table 1:** Morbidity and mortality rates during the Bluetongue virus serotype1 outbreak in Basque Country.

Year		Sheep	Cattle	Goats
**2007**	**Morbidity (%)**	1.74	2.28	6.56
	**Mortality (%)**	0.75	0.55	3.28
**2008**	**Morbidity (%)**	2.84	2.10	6.10
	**Mortality (%)**	1.68	0.36	2.56

After confirmation of the first clinical outbreaks, an epidemiological survey was carried out on involved farms. Overall, farms investigated were small with sheep and cattle and sometimes goats. Forty-six surveys were made on sheep farms, of which more than a half were small (fewer than 100 animals), extant (not transhumant to mountain pastures), and often in contact with other neighbor herds. When the survey was carried out, 70% had had at least one dead sheep showing BT clinical signs. Sixteen surveys were carried out on cattle farms where the maximum number of clinically affected animals was two of which none died.

**Figure 2:**
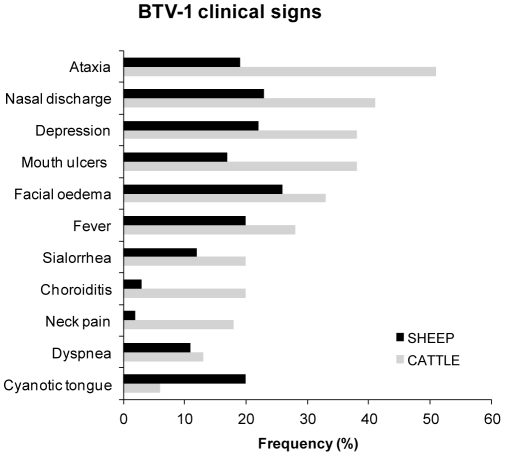
Clinical signs associated to BTV-1 infection in the 2007–2008 outbreaks in Basque Country.

The main clinical signs detected in BTV-1 infected animals diverged depending on the species (Figure 2). Infected sheep showed, in order of importance, facial edema, nasal discharge, depression, cyanotic tongue, ataxia and fever as major clinical signs. In cattle, the most important clinical manifestations were ataxia, nasal discharge, depression, mouth ulcers and facial edema.

### Hypothesis of BTV-1 introduction and spreading into Basque Country

**Figure 3:**
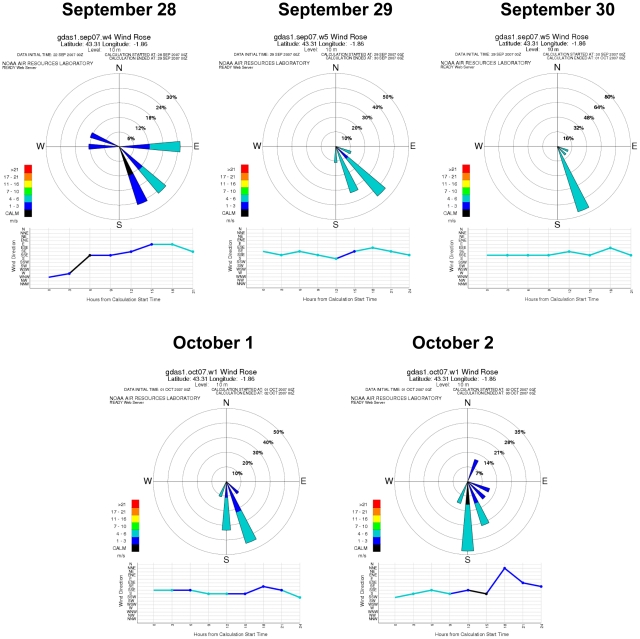
Wind direction and speed in Oiartzun between September 28 and October 2, 2007. Wind rose diagrams show the frequency of wind direction at a single location (Oiartzun) on a 16-point compass. In addition, rings represent the wind speed frequency for seven wind speed classes identified by color. Along the bottom of the plot is a graph of the wind direction versus model forecast hour to give information on when the winds will be from each sector. The color of the line indicates the wind speed at that forecast hour using the same color bands as in the wind rose.

**Figure 4:**
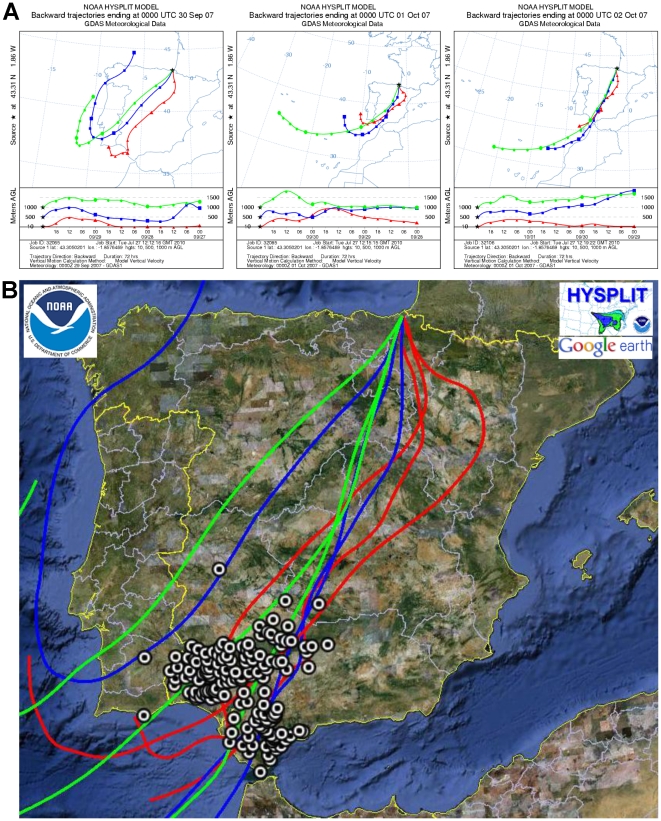
HYSPLIT backward trajectories in Oiartzun and BTV-1 outbreaks in the Iberian Peninsula. A. Backward trajectories from the HYSPLIT model at 10 m (red), 500 m (blue) and 1000 m (green) above ground level. Days: September 30, October 1 and October 2, 2007. B. Dots in the map show the outbreaks in the Iberian Peninsula from August 25 to October 5, before and during the hypothetical wind-borne transport of infected *Culicoides*.

The first clinical outbreak of BTV-1 in the Basque Country was recorded in Oiartzun on November 2, 2007. Considering a minimum period of 4 weeks for the transport of infected midges, spread, infection of new hosts, development of viremia and detection of clinical cases [23], [26], [27] and after analyzing the direction and frequency of predominant winds, we found that since mid-summer, five periods of southerly winds (July 13 to 16, August 25 to 28, September 7, September 16, September 21 and September 28 to October 2, 2007) could be considered as opportunities for midges to have been airborne carried from current infected areas in the South of the Iberian Peninsula (Figure 3). The analysis of backward trajectories for these dates showed September 28 to October 2 as the most probable days for the introduction of infected midges to the Basque Country, assuming that there was not an excessive lag between virus arrival and disease detection and taking into account the known BT incubation period. These wind trajectories crossed through the areas affected by serotype 1 on those dates in the South of the Iberian Peninsula (Figure 4). Besides, meteorological data all along backward trajectories showed suitable conditions for *Culicoides* survival (Table 2).

**Table 2:** Meteorological data along the backward trajectories of winds.

		Height above ground level		
		10 m	500 m	1000 m
**Trajectory 2007 Sep 30**	Air Temperature (°C)	14.4±1.8	10.5±2.8	8.2±2.0
	Relative Humidity (%)	75.5±7.6	86.3±6.8	73.1±12.3
	Rainfall (mm/h)	0.07±0.13	0.1±0.2	0.07±0.18
**Trajectory 2007 Oct 1**	Air Temperature (°C)	13.9±4.2	13.6±1.0	13.8±3.4
	Relative Humidity (%)	71.6±15.2	80±11.8	69.8±14.7
	Rainfall (mm/h)	0.01±0.02	0.02±0.06	0.01±0.03
**Trajectory 2007 Oct 2**	Air Temperature (°C)	16.9±2.3	15.1±1.9	12.5±2.8
	Relative Humidity (%)	67.8±10.4	66.7±7.3	77±3.4
	Rainfall (mm/h)	0.02±0.05	0.02±0.05	0.03±0.07

Between September 29 and October 2, 2007, there was a period of high temperatures (average of 19±1.4°C), zero or very little rain, relative humidity about 76% and southerly winds (147–180° and 5.6 to 9.25 m/s) (Jaizkibel weather station) [24] which represented a great opportunity for *Culicoides* dispersion.

Since only the contiguous Renteria, Hernani and Oiartzun municipalities had herds with a 100% seroprevalence we suspected that infection could have had a longer time-course of infection. Furthermore, the proportion of herds with, at least, 50% seropositive animals in these three municipalities (12/31) was significantly (P<0.001) higher compared to the rest of municipalities that presented clinical cases (4/73). This fact could further set the point of origin of the outbreak in these areas. Although the above statement should be considered with caution because of the low number of affected herds (Table 3).

**Table 3:** Serological data of the initial outbreak locations.

Location	Seroprevalence					No. Herds
	0%	25%	50%	75%	100%	
**AIZARNAZABAL**	50%	50%	0%	0%	0%	2
**ANDOAIN**	50%	50%	0%	0%	0%	4
**ASTIGARRAGA**	17%	67%	17%	0%	0%	6
**DONOSTIA**	43%	43%	14%	0%	0%	7
**RENTERIA** * †	**0%**	**50%**	**17%**	**17%**	**17%**	**6**
**HERNANI** *	**40%**	**20%**	**0%**	**20%**	**20%**	**5**
**HONDARRIBIA**	33%	67%	0%	0%	0%	3
**IDIAZABAL**	0%	0%	0%	100%	0%	1
**IRUN**	25%	75%	0%	0%	0%	4
**OIARTZUN** *	**30%**	**35%**	**10%**	**5%**	**20%**	**20**
**PASAIA**	0%	100%	0%	0%	0%	1
**USURBIL**	50%	50%	0%	0%	0%	2
**VILLABONA**	0%	0%	0%	100%	0%	1

* Bold rows indicate the locations with a seroprevalence of 100% in some herd.

† Specifically, Rentería showed serological response in all analyzed herds.

In addition, we checked cattle movement records in Gipuzkoa during the period immediately previous and following the initial outbreak and there was no cow imported from the affected areas, nor birth of calves that could be related to an import of virus in cattle. Also, it is noteworthy to point out that at those dates there had not been any bluetongue outbreak in the regions between southern Spain and the Basque Country, which rules out any small jumps of the virus by local midge movements between the regions.

## Discussion

*Culicoides* midges seem to have two types of flight, short distance flights (1–2 km) that occur in any direction and at low or zero wind speeds; and long distance flights (up to 700 km), where midges are mainly passively carried by the wind because of their small size [8], [28].Usually adult midges make “swarms” for reproduction that could be elevated tens of meters above ground by air updrafts under specific temperature conditions. Generated airflows with a speed about 3–11 meters per second, a temperature less than 30°C and a relative humidity above 25% could carry these insects alive hundreds of miles [29].

Some investigations show that long distance movements are not accidental but are actively initiated and maintained [30].These movements may be finished either actively (by the insect ceasing to move its wings and descending), because the wind drops or when warm air meets cold air from different direction (fronts may lead to convergence and concentration of the insects) [28], or due to terrain topography [31], [32]. After landing, the midge must survive long enough to replicate the virus, and to bite a susceptible ruminant host. The probability is influenced by the local habitat, the weather conditions, the hosts at destination and the presence or absence of the virus at the source [23], [26]. The transport of BTV-infected *Culicoides* on the wind has been implicated as the most likely source of introduction of BTV in some previous outbreaks [33]–[35], as well as of other insect-borne viruses [21], [36], [37].

The HYSPLIT model was chosen because it is readily available, does not require extensive data processing, has been extensively used in other applications [18]–[21], [38], [39] and seemed to suit best the epidemiological objectives of our study focused on testing a hypothesis of wind long range transport over land. This model has some limitations related to the low vertical and horizontal resolution of the meteorological data and to the use, in our case and for the sake of simplicity, of only 3 trajectories to represent the stochastic, turbulent motions of the atmosphere. Additionally, lack of inclusion of land physiography might further decrease the accuracy of surface trajectories. Other models that have been used to assess the risk of windborne dispersal of *Culicoides* are the NAME III and the MATCH. The first was used by Gloster *et al*., to determine the high risk periods of windborne transport of *Culicoides* midges from Belgium to the UK, and by Burgin *et al*. to Norway [40],[41]. The MATCH model was used by Persson and Agren *et al*. in Sweden [26],[42]. Both models have a higher resolution although they are built on different mathematical principles and use different sources of meteorological data and resulted more difficult to implement with our specific data and resources. Other epidemiological tools were designed for scenario modeling by Szmaragd *et al*. and Ducheyne *et al*. [43], [44]. Thus, while the use of these different models for the study of vector-borne diseases helps to understand the workings of the natural phenomena they try to describe, an agreement might be necessary for the development of a veterinary application that is open to the scientific community and to risk managers both in terms of tools and data and that accounts for the specificities of the most common vector borne diseases for improved decision-making and control strategies design.

Current knowledge of the Bluetongue serotype 1 transmission held that it is linked to the presence of *Culicoides imicola*, which had not ever been detected at these latitudes [7], [45]. This fact could be due to a lack of sampling, because until the first outbreak there was only a trap installed in Araba within the framework of the National Bluetongue Entomological Surveillance Program. After the first outbreak the trapping was extended to the three provinces. This trapping effort from November 2007 and January 2008 yielded a total of 43,051 *Culicoides* specimens. Six specimens of *Culicoides imicola* were detected in both of the localities most affected by the outbreak of BTV-1 [13].

Considering the geographical distribution of the different outbreaks, it was proposed that the most likely scenario was the arrival of BTV infected midges (*Culicoides imicola*) from warm air masses from the south of the Iberian Peninsula. That preliminary conclusion would be supported by the absence of this species outside the dates with BTV-1 outbreaks [13]. This can be explained by the disappearance of these species colonies in the winter period in addition to the extensive coverage of sheep vaccination before the spring that probably stop clinical outbreaks.

After the initial outbreaks, it seems a series of outbreaks occurred that did not necessarily involve a new arrival of infected *Culicoides.* The virus could pass the winter and early spring in cattle that act as a reservoir and then have experienced low-distance spread with the participation of other vectors like *Culicoides obsoletus*, *C. pulicaris, C. lupicaris* or *C. nubeculosus* as had been suggested in the BTV-8 outbreaks of northern Europe [46].

After two months without new cases (June and July, 2008), new outbreaks emerged again in early August 2008 in cattle in Bizkaia, again caused by serotype 1. Delayed appearance of new outbreaks of serotype 1 might suggest a new virus arriving from the south that would have affected unprotected animals. However, this hypothesis would be rather unlikely because in 2008 there had only been BTV-1 outbreaks reported in southern Portugal, but not in southern Spain. Besides, several BTV serotype 1 outbreaks were reported in the nearby regions of Asturias and Cantabria during this period. Alternatively, more efficient transmission by midge species more active during fall months than during summer might account for these late outbreaks.

The absence of these arrival pathways in serotype 4 introduces a contradictory element to this model; and that lead us to postulate that perhaps, the transmission paths could be different for each serotype. Alternatively, there are questions about whether there have been no change in the virus-vector-host relations that had occurred in recent years after gaining apparent control of serotype 4 in southern Spain. This possibility, which also could explain the spread of serotype 8 in exceptionally high northern latitudes, is most disconcerting because it opens the way for the entry of successive epidemics in the future.

Surprisingly, the high incidence of the infection in cattle described in the Basque Country outbreaks was not reported in the southern epidemics during 2007, where neither clinical signs nor mortality were observed in cattle herds [47]. This fact would require a deeper investigation but could be preliminarily associated to the different breeds, management, and cattle production systems used in both territories. Another important difference could be the previous vaccination against BTV-serotype 4 carried out in the southern cattle that could provide cross-protection to BTV-serotype 1.

This analysis suggests that transmission of each serotype might depend on different mechanisms/vectors. Thus, if confirmed, it would imply that every BTV serotype should be treated differently from an epidemiological point of view. Obviously, subsequent control measures should also be different. For example, the management of serotype 1 should be based on vaccination and control in the origin infected areas, while management of serotype 8 should be centered on imports control. The logistic and economic consequences can be tremendously different.

Unfortunately, serotypes 1 and 4 have been detected again in Morocco, Tunisia and Algeria between October 2009 and July 2010. Thus, the last risk analysis of the Spanish authorities considered the highly possibility that new infective vectors from North Africa could reach the South of the country from May to December 2010, as had been confirmed with the detection of BTV serotypes 1, 4 and 8 circulation in the Southern territories of the Iberian Peninsula in September and October, 2010. Taking into account this situation, preventive vaccination against BTV serotype 4 had been applied in the South of Spain, which involved the consideration of this area as “BTV-1,4,8 Restricted Zone” [48].

In conclusion, **s**omething might be changing in the geographical distribution of vector-borne diseases. The specific mechanisms of BT spreading are currently under investigation. These should include active virological and entomological surveillance as well as clinical and serological follow-up. The speed, depth and consequences of new BTV infections are very difficult to predict. However, the recent spread of BTV-1 in Northern Spain or BTV-8 in Northern Europe could provide a warning signal, as the “tip of the iceberg” of other arbovirus infections waiting to emerge.
